# Development and Validation of Transformer- and Convolutional Neural Network-Based Deep Learning Models to Predict Curve Progression in Adolescent Idiopathic Scoliosis

**DOI:** 10.3390/jcm14207216

**Published:** 2025-10-13

**Authors:** Shinji Takahashi, Shota Ichikawa, Kei Watanabe, Haruki Ueda, Hideyuki Arima, Yu Yamato, Takumi Takeuchi, Naobumi Hosogane, Masashi Okamoto, Manami Umezu, Hiroki Oba, Yohan Kondo, Shoji Seki

**Affiliations:** 1 Department of Orthopaedic Surgery, Osaka Metropolitan University, 1-4-3 Asahimachi Abeno-ku, Osaka 545-8585, Japan; stakahashi@omu.ac.jp; 2 Department of Radiological Technology, Graduate School of Health Sciences, Niigata University, 2-746 Asahimachi-dori, Chuo-ku, Niigata 951-8518, Japan; ichikawa@clg.niigata-u.ac.jp (S.I.); mokamoto@clg.niigata-u.ac.jp (M.O.);; 3 Niigata Spine Surgery Center, Kameda Daiichi Hospital, Niigata 950-0165, Japan; 4 Department of Orthopaedic Surgery, Dokkyo Medical University, 880 Kitakobayashi, Shimotsuga, Mibu 321-0293, Japan; ueharu@dokkyomed.ac.jp; 5 Department of Orthopaedic Surgery, Shinshu University School of Medicine, Hamamatsu 431-3192, Japan; arima@hama-med.ac.jp (H.A.); yamato@hama-med.ac.jp (Y.Y.); 6 Department of Orthopaedic Surgery, Kyorin University, Tokyo 181-8611, Japan; kimotako1107@yahoo.co.jp (T.T.);; 7 Department of Orthopaedic Surgery, Shinshu University School of Medicine, Matsumoto 390-8621, Japan; 8 Department of Orthopaedic Surgery, Faculty of Medicine, University of Toyama, 2630 Sugitani, Toyama 930-0194, Japan

**Keywords:** adolescent idiopathic scoliosis, convolutional neural network, artificial intelligence, prediction, curve progression

## Abstract

**Background/Objectives**: The clinical management of adolescent idiopathic scoliosis (AIS) is hindered by the inability to accurately predict curve progression. Although skeletal maturity and the initial Cobb angle are established predictors of progression, their combined predictive accuracy remains limited. This study aimed to develop a robust and interpretable artificial intelligence (AI) system using deep learning (DL) models to predict the progression of scoliosis using only standing frontal radiographs. **Methods**: We conducted a multicenter study involving 542 patients with AIS. After excluding 52 borderline progression cases (6–9° progression in the Cobb angle), 294 and 196 patients were assigned to progression (≥10° increase) and non-progression (≤5° increase) groups, respectively, considering a 2-year follow-up. Frontal whole spinal radiographs were preprocessed using histogram equalization and divided into two regions of interest (ROIs) (ROI 1, skull base–femoral head; ROI 2, C7–iliac crest). Six pretrained DL models, including convolutional neural networks (CNNs) and transformer-based models, were trained on the radiograph images. Gradient-weighted class activation mapping (Grad-CAM) was further performed for model interpretation. **Results**: Ensemble models outperformed individual ones, with the average ensemble model achieving area under the curve (AUC) values of 0.769 for ROI 1 and 0.755 for ROI 2. Grad-CAM revealed that the CNNs tended to focus on the local curve apex, whereas the transformer-based models demonstrated global attention across the spine, ribs, and pelvis. Models trained on ROI 2 performed comparably with respect to those using ROI 1, supporting the feasibility of image standardization without a loss of accuracy. **Conclusions**: This study establishes the clinical potential of transformer-based DL models for predicting the progression of scoliosis using only plain radiographs. Our multicenter approach, high AUC values, and interpretable architectures support the integration of AI into clinical decision-making for the early treatment of AIS.

## 1. Introduction

Despite ongoing clinical and genetic investigations, the etiology of adolescent idiopathic scoliosis (AIS) remains poorly understood [1]. A significant challenge in the management of AIS is the unpredictability of curve progression. Although factors such as initial curve magnitude and skeletal maturity are the most recognized predictors [2], additional elements—including age, menarcheal status, bone mineral density, and curve morphology—may contribute to the risk of progression [2,3,4]. However, even when these factors are considered together, their combined predictive power is limited and methods for accurately forecasting an individual patient’s curve progression remain elusive [5,6]. This uncertainty in prognosis can complicate management decisions and underscores the need for more reliable predictive tools.

Artificial intelligence (AI), particularly deep learning (DL), has shown strong potential for analyzing complex patterns within medical images [7]. A prominent DL approach for images is the convolutional neural network (CNN), which automatically learns to recognize visual features that are relevant to a given task [8]. CNNs have been successfully applied for the detection and classification of various musculoskeletal disorders [9,10,11]. The field of orthopedics has shifted toward a paradigm of predictive prevention, emphasizing the early identification of high-risk patients to prevent disease progression or future structural failure [12]. A previous pilot study [6] applied a CNN-based model to predict curve progression in AIS patients and achieved a moderate area under the curve (AUC) of 0.70. However, that study was limited by a small sample size, the use of only one type of model, and lack of validation using external data. Furthermore, traditional CNNs are inherently limited by their focus on local features in images, which may restrict their ability to capture global spinal deformity patterns [13]. In AIS radiographs, important indicators of progression risk might be encoded in the overall spinal alignment and curve shape, spanning from the upper thoracic spine and ribs down to the lumbar spine and pelvis.

New AI architectures, including vision transformers, have recently been developed to overcome some of the limitations of CNNs in image analysis. A vision transformer (ViT) includes an attention mechanism that allows the model to analyze all parts of an image collectively [14,15]. This global perspective is well-suited in the context of scoliosis, where the pattern of curvature from the upper thoracic to the lower lumbar spine—including the pelvis—may provide important clues about the risk of progression. Previous studies have highlighted that ribcage indices and pelvic parameters may serve as structural predictors of curve progression [16,17]. ViTs are theoretically capable of recognizing these distributed features simultaneously, enabling a more comprehensive assessment of progression risk. By capturing the full-spine deformity pattern, transformer-based models might achieve improved prediction accuracy.

In this multicenter study, we aimed to develop a generalizable, interpretable, and high-performance AI system for predicting the scoliosis curve progression using only standard frontal radiographs. We leveraged state-of-the-art ViT models along with a diverse, multicenter radiograph dataset, in order to ensure that the models learn a broad range of curve patterns and do not overfit to a single population. Furthermore, we incorporated attention-based interpretability techniques to create transparent models that highlight the image regions which most strongly influence their predictions. Such interpretability is essential for building clinical trust in AI-driven tools, enabling clinicians to understand why the model predicts a high risk of progression for a given patient. This multicenter study aimed to provide a robust and transparent decision-support tool which can identify high-risk AIS patients early on, thus facilitating personalized treatment planning and potentially improving patient outcomes.

## 2. Materials and Methods

### 2.1. Study Design and Setting

This multicenter, observational study was conducted at six academic institutions in Japan. Both retrospective and prospective patient cohorts were included. The study protocol was approved by the institutional review boards of all the participating institutions and adhered to the ethical standards outlined in the Declaration of Helsinki.

### 2.2. Patient Enrollment

For the retrospective arm, patients diagnosed with AIS of at least 10° who visited the participating hospitals between January 2010 and the ethical approval date were identified. The inclusion criteria were as follows: (1) age ≥ 10 years, (2) a confirmed diagnosis of AIS based on clinical and radiographic assessment, and (3) the availability of standing frontal whole-spine radiographs at baseline and after 2 years. For the prospective arm, patients were enrolled during their first outpatient visit after obtaining informed consent. The exclusion criteria included congenital, syndromic, or neuromuscular scoliosis and cognitive impairment that interfered with informed consent or clinical evaluation.

### 2.3. Conservative Treatment

Brace treatment was recommended for skeletally immature patients with Cobb angles ≥25°, particularly those with Risser sign 0–2 and Cobb angle between 25° and 40°. Bracing was specifically indicated for premenarcheal patients or those within 1 year of menarche. The discontinuation of bracing was considered after skeletal maturity was attained and no significant progression was noted, with a gradual reduction in daily wear time.

### 2.4. Data Collection

Data regarding demographics, treatment status, and radiographic measurements were collected at baseline. Standing whole-spine frontal radiographs were acquired at baseline and the 2-year follow-up. These images were then exported from the institutional PACS systems and saved in standard PNG file format. All images were anonymized and standardized for the analysis.

### 2.5. Prediction Task and Ground Truth Definition

The model was trained to predict the progression of scoliosis over 2 years from only baseline standing frontal radiographs. Progression was defined as a ≥10° increase and non-progression as a ≤5° increase in the Cobb angle between the baseline and follow-up images. These labels were used as the ground truth for supervised learning.

### 2.6. Imaging Preparation and Preprocessing

**Figure 1:**
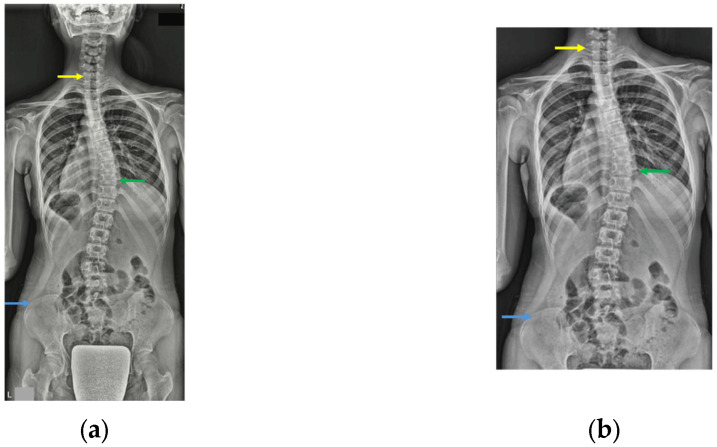
Image input regions of interest (ROIs). Yellow, green, and blue arrows show C7 vertebra, apex of main thoracic curve, and iliac crest, respectively. Two ROIs were defined: ROI 1 included the full frontal image from the skull base to the femoral head (**a**), and ROI 2 was cropped from C7 to the iliac crest (**b**).

Two regions of interest (ROIs) were defined: ROI 1 included the full frontal image from the skull base to the femoral head, while ROI 2 was cropped from C7 to the iliac crest (Figure 1). Given that the skull base and femoral heads are sometimes absent in standing whole-spine radiographs, ROI 2 (C7–iliac crest) was defined to reflect these common imaging variations, as well as to assess the model’s robustness and generalizability when trained on reduced anatomical information. Preprocessing included contrast-limited adaptive histogram equalization (CLAHE), zero-padding along the shorter side to produce square images, and pixel intensity normalization using the mean and standard deviation of the pretrained models. All images were subsequently rescaled to an intensity range of [0, 1] before serving as the model input. Data augmentation was applied in an online manner, and only to training images. This included random horizontal flipping with a probability of 0.5 and random cropping to the model input size, with a cropping scale ranging from 50% to 100% of the original image area and a fixed aspect ratio of 1:1.

### 2.7. Deep Learning Model Architectures

Six state-of-the-art DL models were selected as backbones for evaluation: ResNet50, DenseNet121, InceptionV3, ConvNeXtV2, ViT-B/16 [18], and SwinT-B [19]. Three models (ResNet50, DenseNet121, and InceptionV3) commonly extract features by scanning small regions of the image, while the other three (ConvNeXtV2, ViT-B/16, and SwinT-B) are based on the newer transformer architecture, allowing the models to analyze the entire image at once and, thus, better capture global patterns such as overall spinal alignment. All models were pretrained on the ImageNet dataset and fine-tuned using the collected scoliosis dataset. A shared classification head was appended to the output of each backbone, comprising LayerNorm, a linear layer projecting the number of feature dimensions to 512, GELU activation, dropout (*p* = 0.5), and a final linear layer outputting the two classes. The input image dimensions were standardized according to the input requirements of each pretrained model: 224 × 224 pixels for ResNet50 and DenseNet121, 299 × 299 pixels for InceptionV3, and 384 × 384 pixels for the ConvNeXtV2 and transformer-based models. All training was performed using PyTorch Lightning v2.5.0 on a system equipped with an Intel Core i9-3900 processor (Intel Corporation, Santa Clara, CA, USA) and a GeForce RTX 4090 GPU (Nvidia Inc., Santa Clara CA, USA).

### 2.8. Model Training and Validation

**Figure 2:**
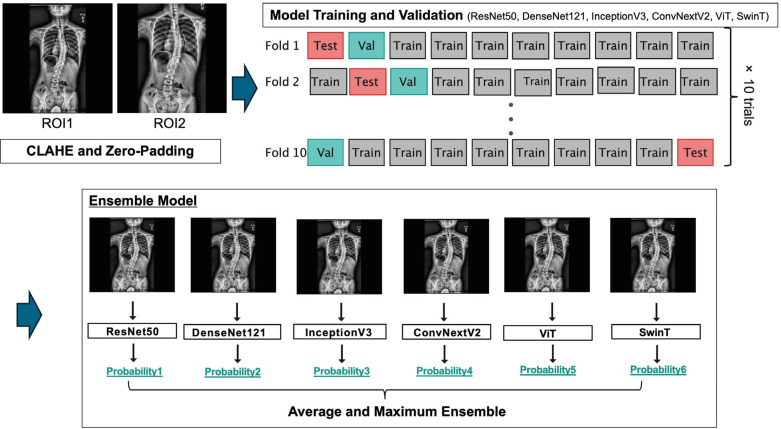
Flowchart of the deep learning framework, showing the schematic of the deep learning pipeline used in this study. After image preprocessing with contrast-limited adaptive histogram equalization and zero-padding, radiographs from the two defined regions (region of interest [ROI] 1 and ROI 2) were input into six deep learning models (ResNet50, DenseNet121, InceptionV3, ConvNeXtV2, ViT, and SwinT). Training and validation were performed using repeated stratified 10-fold cross-validation to ensure robustness and generalizability.

To ensure the model’s robustness, we implemented a stratified 10-fold cross-validation that was repeated 10 times (100 runs per model) (Figure 2). The data were partitioned into training (80%), validation (10%), and testing (10%) sets. For training, the AdamW optimizer (learning rate = 1 × 10^−5^, weight decay = 1 × 10^−3^) with a batch size of 32 and weighted binary cross-entropy loss were used. The class weights were determined based on the inverse frequency of each class in the training set. A cosine annealing learning rate schedule with a five-epoch warm-up and early stopping based on the validation loss after five non-improving epochs was applied. The maximum number of training epochs was set to 100.

### 2.9. Model Interpretability

To assess their explainability, we applied gradient-weighted class activation mapping (Grad-CAM) to all the models. Grad-CAM generates heatmaps that highlight the most influential image regions that contribute to a model’s prediction. We averaged the Grad-CAM outputs across 10 runs to improve the robustness of the resulting heatmaps.

To quantitatively evaluate the attention characteristics of the CNN-based models, an additional analysis of Grad-CAM attention maps was performed. For each model, Grad-CAM heatmaps were generated from all test images and normalized to 8-bit grayscale intensity. Mean and standard deviation (SD) maps were then computed across all cases to visualize the average spatial distribution and inter-case variability of attention, respectively. A higher internal SD value indicates concentrated, localized activation with distinct bright and dark regions, whereas a lower internal SD represents a more diffusely distributed and globally uniform attention pattern across the entire image.

### 2.10. Statistical Analyses

The patients were categorized based on the change in Cobb angle observed at the 2-year follow-up: non-progression (≤5°), borderline (6–9°), and progression (≥10°). The borderline group was excluded from model training and evaluation due to the inherent variability in manual Cobb angle measurements [20]. Model performance was evaluated using multiple metrics, including AUC, sensitivity, specificity, positive predictive value (PPV), negative predictive value (NPV), accuracy, and F1 score. The AUC was calculated as the integral of the true positive rate plotted against the false positive rate, across all classification thresholds [21]. The performance metrics were defined as follows: Accuracy = (True Positives + True Negatives)/(True Positives + True Negatives + False Positives + False Negatives); Sensitivity (Recall) = True Positives/(True Positives + False Negatives); Specificity = True Negatives/(True Negatives + False Positives); PPV = True Positives/(True Positives + False Positives); NPV = True Negatives/(True Negatives + False Negatives); F1 Score = 2 × (Precision × Recall)/(Precision + Recall), where Precision = True Positives/(True Positives + False Positives) [22]. AUC scores were computed using 10-fold cross-validation and are reported as the mean ± standard deviation over 10 repeated runs, enabling better assessment of model stability and robustness.

To further enhance the prediction performance, we explored two ensemble strategies using the predicted probability for the progression class: (i) the average ensemble, which computes the mean of the predicted probabilities across the models; and (ii) the max ensemble, which uses the maximum predicted probability among the six models. The thresholds were optimized using Youden’s indices. For statistical comparisons between models, we applied the Friedman test followed by the Nemenyi post hoc test to assess significant differences between the AUC values across multiple models.

As a comparative baseline, we additionally constructed a traditional logistic regression model using the baseline Cobb angle, age, sex, and Risser sign as input features. This model also aimed to predict curve progression over 2 years. We applied the same repeated 10-fold cross-validation framework used for the DL models to ensure a fair comparison.

To assess the reliability of Cobb angle measurements, we performed both inter-rater and intra-rater reliability analyses. Cobb angle measurements were independently performed on a subset of 40 radiographs by two raters and re-measured by the same raters after a time interval of 3 months. The inter- and intra-rater reliabilities were evaluated using intraclass correlation coefficients (ICC). According to established guidelines, ICC values less than 0.50 indicate poor reliability, values between 0.50 and 0.75 indicate moderate reliability, values between 0.75 and 0.90 indicate good reliability, and values greater than 0.90 indicate excellent reliability [23]. Statistical analysis was performed using R (version 4.4.1).

## 3. Results

### 3.1. Patient Characteristics

In total, 542 patients with AIS were included in this study. After excluding 52 borderline cases, 294 and 196 patients were classified into the progression and non-progression groups, respectively. Table 1 shows the baseline characteristics of the patients. The mean ages at baseline were 12.6 ± 1.9 years in the progression group and 12.9 ± 1.9 years in the non-progression group (*p* = 0.066). Patients with curve progression had a significantly greater initial Cobb angle (mean, 30.8° ± 10.6°) than those without progression (mean, 23.8° ± 8.2°), (*p* < 0.001). Regarding baseline curve severity, 205 patients with Cobb angles of 10–24° and 290 patients with Cobb angles ≥ 25° were included. Among patients with 10–24° curves, 71 (34.6%) experienced curve progression, compared to 227 (78.3%) in the ≥25° group (*p* < 0.001). No significant differences were observed in terms of sex, height, weight, or menarcheal status. The Risser sign distribution indicated greater skeletal immaturity in the progression group (*p* = 0.011). Regarding the curve pattern, thoracic curve was more frequently distributed in the progression group (*p* = 0.012), with left thoracic curve observed in 10 patients (10/310 = 3.2%). The inter-rater ICC was 0.966, indicating excellent agreement. The absolute mean difference was 1.55°, with a standard error of 0.19°. The intra-rater ICC between Cobb was 0.933, also indicating excellent reliability. The absolute mean difference was 2.00°, with a standard error of 0.29°. The logistic regression model based on the baseline Cobb angle, age, sex, and Risser sign achieved an average AUC of 0.726 ± 0.005 across repeated 10-fold cross-validation.

### 3.2. Model Performance with ROI 1 (Full Image)

**Figure 3:**
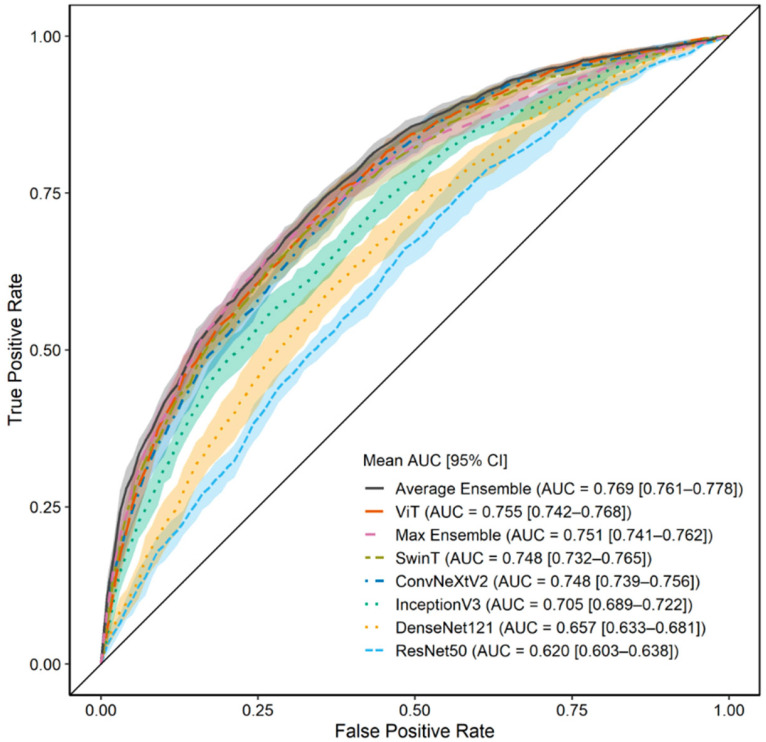
Prediction results when using region of interest (ROI) 1 (full image). The area under the curve (AUC) distribution across 10 repeated trials is shown for each of the six individual models and the ensemble models trained on ROI 1. The accompanying plot displays the average receiver operating characteristic (ROC) curve with shaded 95% confidence intervals, indicating the classification performance for scoliosis progression prediction using full frontal radiographs.

We first evaluated the model performance using ROI 1. As shown in Table 2, among the six individual models, the ViT achieved the greatest average AUC of 0.755 ± 0.021, followed closely by SwinT and ConvNeXtV2. The ensemble approach using predictions averaged across all six models showed the best overall performance (AUC, 0.769 ± 0.014). This ensemble model yielded an accuracy of 0.704 ± 0.020 and an F1-score of 0.741 ± 0.033, outperforming each individual model. These findings are graphically represented in Figure 3, where the receiver operating characteristic (ROC) curves of each model are plotted with 95% confidence intervals. The ensemble model consistently demonstrated a superior ROC curve, suggesting that combining model predictions improves robustness and generalization in the context of curve progression classification.

Statistical analyses using the Friedman test, followed by the Nemenyi post hoc test, confirmed significant differences between the models. Notably, ViT performed significantly better than ResNet50 and DenseNet121 (*p* < 0.05), emphasizing the improved prediction capabilities of modern transformer-based and next-generation convolutional networks.

### 3.3. Model Performance with ROI 2 (C7 to Iliac Crest)

**Figure 4:**
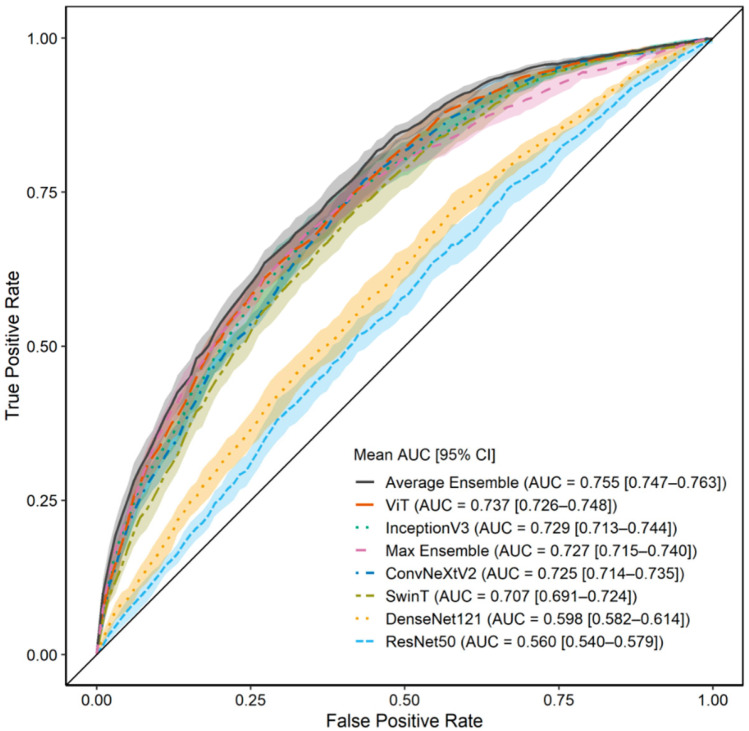
Prediction results using region of interest (ROI) 2 (C7–iliac crest cropped image), summarizing the prediction performance of all models when trained on the ROI 2 input. The AUC distribution across 10 repeated trials is shown, along with the average ROC curve with 95% confidence intervals for each model using ROI 2.

To assess the influence of anatomical region selection, we trained the same models using ROI 2. As shown in Table 3, the model’s performance was slightly worse than that when using ROI 1. ViT continued to outperform the other architectures. The average ensemble for ROI 2 achieved an AUC of 0.755 ± 0.013, comparable with that for ROI 1, with an accuracy of 0.700 ± 0.025 and F1-score of 0.737 ± 0.044. Figure 4 shows the corresponding ROC curves for the models trained using ROI 2. Although the AUC when using ROI 2 was slightly less than that for ROI 1, the performance remained robust, indicating that the cropped images focusing on the spine retained sufficient predictive information [24]. As with ROI 1, ViT was significantly superior to ResNet50 and DenseNet12 (*p* < 0.05), confirming that transformer-based architectures generalize well across the input region definitions.

### 3.4. Gradient-Weighted Class Activation Mapping Attention Analysis

**Figure 5:**
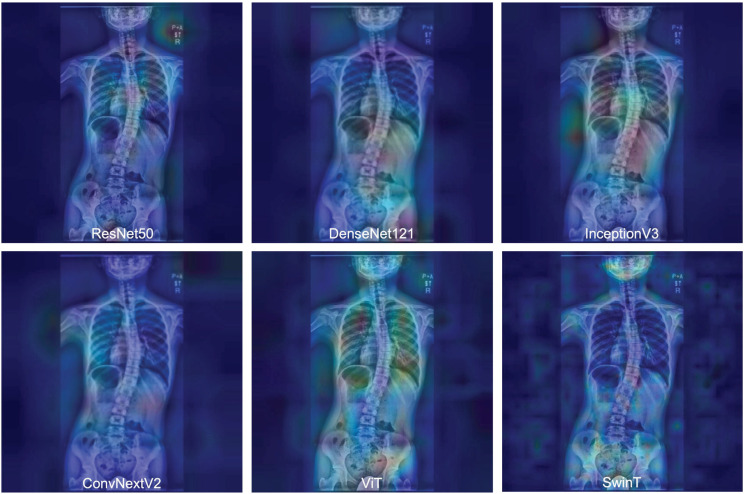
Grad-CAM attention heatmaps by model architecture, with representative Grad-CAM heatmaps shown for the six deep learning models trained on ROI 1 inputs. The heatmaps highlight the key regions contributing to the predictions, varying across models from the thoracic and lumbar spine to non-spinal areas. Each image reflects the average attention map across 10 repeated trials per model.

Representative activation maps are shown in Figure 5. In correctly predicted progression cases, CNN-based models—such as ResNet50, DenseNet121, and InceptionV3—focused on regional anatomical features, particularly the apex of the spinal curve in the thoracic and lumbar spines. This targeted attention pattern supports the role of localized feature extraction in CNN architecture.

In contrast, transformer-based models—such as ViT and SwinT-B—exhibited more distributed activation across the entire image, including spinal alignment, rib contours, and pelvic tilt. This broader and holistic focus suggests that models based on transformer architectures capture global contextual information beyond the focal curvature region.

Specifically, the internal SD values of the mean Grad-CAM maps were as follows: ResNet50 (17.2), DenseNet121 (21.2), InceptionV3 (20.0), ConvNeXtV2 (20.7), ViT (6.0), and SwinT (16.9). These findings quantitatively support the qualitative observation that CNNs focus on regional features, whereas transformer-based models analyze global structural patterns encompassing the entire spine.

## 4. Discussion

Curve progression in AIS patients remains a critical concern in clinical practice, as the early identification of patients at high risk is essential for timely intervention. Traditional predictive indicators—such as the Cobb angle, skeletal maturity, and Risser sign—are useful but limited, and there remains a gap in accurately forecasting future curve progression using noninvasive tools. DL applied to standard radiographs presents an opportunity to bridge this gap, enabling clinicians to make more informed decisions regarding observation, bracing, or surgical referral. We developed and validated DL models to predict curve progression in idiopathic scoliosis using standing frontal radiographs. Advanced architectures, particularly ViTs, outperformed traditional CNNs across multiple evaluation metrics. The average ensemble strategy achieved superior overall performance, underscoring the value of combining algorithmic perspectives to enhance robustness across different input regions. The predictive capability of our AI model has significant implications for treatment planning in the context of idiopathic scoliosis.

Curve progression in idiopathic scoliosis is multifactorial [2,25]. The initial Cobb angle is a well-established predictor of disease progression [26], as well as skeletal immaturity [27]. Curve morphology is also important, as thoracic and double major curves are more likely to progress [28]. Although less well-studied, vertebral rotation is also a potential predictive factor for progression [29]. Moreover, radiographic cues may reflect the systemic bone mineral density status [30]. The DL model’s task is essentially to evaluate such risk factors from the radiograph alone. The developed models showed stronger performance, compared to a traditional logistic model using just the Cobb angle, age, sex, and Risser sign (AUC = 0.726), with the transformer-based DL models achieving AUCs of 0.755–0.769 depending on the ROI and ensemble strategy. These findings support the applicability of such DL models, even in patients with mild to moderate curves, and emphasize their value in early risk stratification.

Previous studies have shown that deep learning approaches allow for the extraction of meaningful clinical features from spine X-rays. For instance, AI systems have been used to measure spinal alignment, classify curve types, and even detect scoliosis from radiographs [31]. Radiographic data may be used opportunistically to assess patient-specific parameters [32,33]. The superior performance of our models may reflect their ability to implicitly capture the underlying structural indicators without explicit feature engineering. Moreover, traditional metrics typically require expert interpretation; however, our results suggest that modern AI architectures can automatically infer many of these features directly from image data. While the considered models enable estimation of the probability of curve progression, determination at the level of certainty that would influence physician or patient decision-making—such as the decision to initiate bracing—requires further investigation.

The field of AI-based approaches to scoliosis care is rapidly evolving; however, only a few studies have specifically addressed curve progression prediction [6,34,35]. Compared with prior work [6], our study represents a significant advancement in terms of both its scale and methodological rigor. The pilot study was limited by its small sample size, single-center design, and suboptimal image preprocessing. To address these limitations, this study implemented standardized CLAHE, incorporated balanced data from six institutions, and used repeated cross-validation to mitigate sampling bias. Importantly, this study used two ROIs to assess the robustness of the models, thus supporting the utility of a standardized cropping approach to optimize model consistency without sacrificing predictive accuracy. In addition, in a previous study [35] using a modified CapsuleNet architecture, radiographic and clinical data were combined to improve the prediction accuracy; however, this approach relied on EOS bi-planar stereoradiography (EOS^®^ imaging system, Paris, France), thus limiting its applicability to standard radiography settings. Although other machine learning models have been applied in similar contexts, they typically require manual feature selection and do not learn directly from raw images [36]. In contrast, our end-to-end DL design may facilitate easier integration into routine clinical workflows.

A key insight from this study is the comparison between CNN- and transformer-based architectures. CNNs analyze images using fixed-size local regions, limiting their ability to capture broader anatomical context [13]. In scoliosis imaging, where deformity-related features can span from the cervical spine to the pelvis, such localized analysis may miss important global patterns. In contrast, ViTs divide the image into small patches and use self-attention mechanisms to evaluate relationships across the entire image [37], allowing them to recognize the long-range structural patterns that are important for understanding spinal alignment. Our Grad-CAM analysis confirmed this difference, showing that CNNs tended to focus on specific regions (e.g., the apex of the curve), whereas ViTs distributed attention more broadly across the spine, rib cage, and pelvis, thus aligning more closely with the way in which clinicians assess whole-spine deformities.

Despite these strengths, this study has some limitations. First, our models relied solely on imaging data. Although this supports end-to-end automation, future iterations may obtain improved performance through the incorporation of multimodal inputs. Second, we excluded cases with borderline progression, which may have introduced a selection bias. However, this group inherently represents clinical ambiguity, and their exclusion was intended to sharpen the contrast between progression and non-progression cases, potentially improving the specificity and interpretability of the models. Third, transformer-based models require considerable computational resources, which may hinder their real-time application in settings with limited infrastructure. Fourth, although the model was trained on a diverse multicenter cohort, prospective external validation is essential to assess its performance across broader patient populations and clinical environments. Finally, adolescent idiopathic scoliosis is a polygenetic disorder, and conventional biomarkers—such as skeletal maturity, menarcheal status, and curve severity or location—fail to capture the complex genomic mechanisms underlying disease progression [38]. Therefore, incorporating additional data sources beyond those used in this study may be required to improve the prognostic precision of such models.

## 5. Conclusions

In this study, the ability of transformer-based DL models to predict the progression of scoliosis using only frontal radiographs was confirmed. These findings support the integration of AI-assisted tools into routine scoliosis care for early risk stratification and treatment planning. Future studies should focus on adding clinical data, carrying out prospective validation, and refining model deployment pathways for clinical use.

## Figures and Tables

**Table 1:** Comparison of demographic data.

	Non-Progression N = 196	Progression N = 294	*p*-Value
Age (yrs)	12.9 (1.9)	12.6 (1.8)	0.091
Sex (female)	181 (92.3%)	266 (90.5%)	0.369
Height (cm)	153.9 (7.5)	153.5 (9.2)	0.565
Weight (kg)	45.9 (8.1)	44.8 (9.2)	0.293
Menarche			0.269
Not yet	66 (33.7%)	113 (38.4%)	
≤2 yrs	99 (50.5%)	148 (50.3%)	
>2 yrs	31 (15.8%)	33 (11.2%)	
Brace (>12 h/day)	24 (12.2%)	19 (6.7%)	0.040
Risser sign			0.012
Grade 0	48 (24.5%)	119 (40.5%)	
Grade 1	30 (15.3%)	30 (10.2%)	
Grade 2	42 (21.4%)	55 (18.7%)	
Grade 3	19 (9.7%)	27 (9.2%)	
Grade 4	56 (28.6%)	62 (21.1%)	
Grade 5	1 (0.5%)	1 (0.3%)	
Cobb angle (°)	23.8 (8.2)	30.9 (10.6)	<0.001
10–25°	114 (58.2%)	89 (30.2%)	<0.001
>25°	82 (41.8%)	205 (69.8%)	

**Table 2:** Comparison of the AUC, sensitivity, specificity, PPV, NPV, accuracy, and F1-score of each model when using ROI 1.

Model	AUC	Sensitivity	Specificity	PPV	NPV	Accuracy	F1-Score
Average Ensemble	0.769 ± 0.014	0.714 ± 0.079	0.689 ± 0.078	0.778 ± 0.027	0.622 ± 0.043	0.704 ± 0.020	0.741 ± 0.033
ViT	0.755 ± 0.021	0.738 ± 0.079	0.652 ± 0.090	0.764 ± 0.033	0.631 ± 0.046	0.704 ± 0.019	0.748 ± 0.031
Max Ensemble	0.751 ± 0.017	0.668 ± 0.074	0.732 ± 0.065	0.792 ± 0.026	0.599 ± 0.035	0.694 ± 0.022	0.722 ± 0.037
SwinT	0.748 ± 0.026	0.687 ± 0.079	0.695 ± 0.065	0.774 ± 0.023	0.601 ± 0.036	0.690 ± 0.030	0.725 ± 0.045
ConvNeXtV2	0.748 ± 0.014	0.745 ± 0.096	0.637 ± 0.088	0.758 ± 0.028	0.636 ± 0.058	0.702 ± 0.026	0.747 ± 0.043
InceptionV3	0.705 ± 0.027	0.650 ± 0.106	0.670 ± 0.116	0.753 ± 0.039	0.568 ± 0.041	0.658 ± 0.029	0.691 ± 0.051
DenseNet121	0.657 ± 0.039	0.649 ± 0.125	0.603 ± 0.135	0.717 ± 0.040	0.545 ± 0.056	0.631 ± 0.033	0.673 ± 0.058
ResNet50	0.620 ± 0.028	0.655 ± 0.180	0.553 ± 0.150	0.690 ± 0.022	0.543 ± 0.080	0.614 ± 0.052	0.659 ± 0.092

**Table 3:** Comparison of the AUC, sensitivity, specificity, PPV, NPV, accuracy, and F1-score of each model when using ROI 2.

Model	AUC	Sensitivity	Specificity	PPV	NPV	Accuracy	F1-Score
Average Ensemble	0.755 ± 0.013	0.714 ± 0.107	0.679 ± 0.109	0.775 ± 0.035	0.625 ± 0.060	0.700 ± 0.025	0.737 ± 0.044
ViT	0.737 ± 0.018	0.726 ± 0.128	0.644 ± 0.142	0.762 ± 0.044	0.628 ± 0.063	0.693 ± 0.025	0.735 ± 0.051
InceptionV3	0.729 ± 0.025	0.673 ± 0.065	0.680 ± 0.059	0.761 ± 0.023	0.584 ± 0.035	0.676 ± 0.026	0.712 ± 0.035
Max Ensemble	0.727 ± 0.020	0.667 ± 0.083	0.701 ± 0.073	0.772 ± 0.025	0.589 ± 0.038	0.680 ± 0.026	0.712 ± 0.043
ConvNeXtV2	0.725 ± 0.017	0.772 ± 0.093	0.574 ± 0.062	0.732 ± 0.010	0.641 ± 0.071	0.693 ± 0.034	0.749 ± 0.045
SwinT	0.707 ± 0.027	0.715 ± 0.108	0.605 ± 0.095	0.733 ± 0.026	0.598 ± 0.062	0.671 ± 0.035	0.719 ± 0.051
DenseNet121	0.598 ± 0.026	0.650 ± 0.148	0.526 ± 0.153	0.678 ± 0.030	0.510 ± 0.042	0.600 ± 0.035	0.652 ± 0.079
ResNet50	0.560 ± 0.031	0.623 ± 0.191	0.485 ± 0.177	0.647 ± 0.037	0.479 ± 0.056	0.568 ± 0.057	0.620 ± 0.105

## Data Availability

The data utilized in this study are available from the corresponding author upon reasonable request.
